# EEG abnormality as a prognostic factor in cirrhotic patients with Grade III-IV hepatic encephalopathy requiring mechanical ventilation: A retrospective analysis

**Published:** 2021-07-16

**Authors:** Lalita Gouri Mitra, Geeta Rajput, Vandana Saluja, Guresh Kumar

**Affiliations:** ^1^Department of Anaesthesia Critical Care, Institute of Liver and Biliary Sciences, New Delhi; ^2^Department of Biostatistics, Institute of Liver and Biliary Sciences, New Delhi

**Keywords:** hepatic encephalopathy, liver cirrhosis, electroencephalography, intensive care unit

## Abstract

**Background and aim:**

Hepatic encephalopathy is a serious complication that entails liver cirrhosis with a high mortality rate. The Child- Turcotte-Pugh class (CTP class) and model for end-stage liver disease (MELD) score are two important prognostic indicators for cirrhosis, while sequential organ failure assessment (SOFA) is a dynamic score for the assessment of critically ill patients. Patients with liver disease with advanced CTP class and higher MELD scores have poor prognosis. The aim of this study was to evaluate the role of electroencephalography (EEG) in cirrhotic patients requiring ventilator support for hepatic encephalopathy Grade III-IV.

**Methods:**

A retrospective study was conducted on patients admitted to the liver intensive care unit (ICU) of a tertiary teaching institute. EEG records of 92 patients with Grade III-IV hepatic encephalopathy who were admitted between April 2015 and May 2017 to the liver ICU were analyzed. The correlation between EEG findings and 28-day mortality, ICU length of stay, and the number of days on mechanical ventilation was determined.

**Results:**

Seventy-eight of 92 patients (85%) exhibited bilateral slowing EEG pattern, suggestive of encephalopathy. A triphasic pattern was the most common EEG abnormality in 40% (31 of 78) of the patients. Patients with abnormal EEG had a significantly higher MELD score compared to those with a normal EEG (*P*=0.02). There were no significant differences in length of mechanical ventilation between both groups, but an increasing trend was observed in those with abnormal EEG (*P*=0.09).

**Conclusion:**

EEG findings correlate well with severity of disease in critically ill patients with liver disease.

**Relevance for patients:**

EEG has a role in monitoring and prognostication of hepatic encephalopathy in critically ill patients with liver disease.

## 1. Introduction

Hepatic encephalopathy (HE) is a common complication of liver cirrhosis that presents as a spectrum of neuropsychiatric abnormalities, ranging from subtle personality changes to profound coma [1].

HE has a significant impact on the survival of these patients [2-4] which worsens with a higher grade of HE [5].

Electroencephalography (EEG) is known to be a reliable tool for evaluation and diagnosis of HE [6,7]. It provides vital information about the cortical postsynaptic activity that is modulated by both physiological and pathological diencephalic and brain-stem influences. EEG is used to detect metabolic brain dysfunction, as it is extremely sensitive to metabolic and toxic influences. Therefore, it is considered a useful neurophysiologic diagnostic modality in patients with grade III-IV HE [8]. It mainly serves two diagnostic roles in cirrhotic patients with altered consciousness. First, it aids in the detection of subclinical HE and second, it predicts the progression to overt HE [9]. This facilitates the initiation of preventive measures and prognostication [10,11].

Parson-Smith *et al*. first introduced the grading system of HE based on EEG characteristics [12]. Subsequently its role in detecting and monitoring the varying grades of HE was recognized [13].

Van der Rijt *et al*. studied EEG patterns in chronic liver disease patients. The authors concluded that progressive slowing of the mean dominant frequency of EEG was associated with a higher grade of HE. Grade I HE exhibited prominence of theta waves, whereas in Grade IV HE delta waves were more prominent. It also depicted a significant correlation between the grade of HE and survival [14].

At present, there is limited understanding of EEG findings in advanced HE patients requiring mechanical ventilation and how these findings influence the overall outcome. Therefore, a retrospective study was conducted with the aim to analyze EEG findings in patients with HE grade III-IV requiring mechanical ventilation, and their association with 28-day survival. The secondary objectives were to analyze the correlation of abnormal EEG patterns with the length of ICU stay and duration of mechanical ventilation.

## 2. Methods

We retrospectively analyzed EEG records of 92 patients admitted with HE Grade III-IV, requiring mechanical ventilation, between April 2015 and May 2017.

We have a 16 bedded intensive care unit dedicated to critically ill patients with liver disease. Patients who required intubation and mechanical ventilation for reasons other than HE were excluded. As per the institutional protocol, EEG analysis was performed at least 24 h after discontinuing sedation, and ruling out other confounding factors. All patients underwent brain imaging to rule out intracranial pathology and received anti coma measures such as purging of the gut, Rifaximin, L-ornithine L-aspartate (LOLA), and organ support as per standard ICU protocol.

Data were collected from electronic medical records (Hospital Information System), for patients who fulfilled the following inclusion criteria,

1. Patients with liver cirrhosis
2. Age >18 years
3. Mechanical ventilation in view of encephalopathy
4. Availability of EEG analysis, 24 h of cessation of sedation.

Demographic characters of age, sex, and etiology of liver disease were noted. The sequential organ failure assessment (SOFA), Child-Turcotte-Pugh (CTP) class, and model for end-stage liver Disease (MELD) score at admission to ICU and SOFA score on the day of EEG analysis were recorded. A twenty channel EEG was performed on Nihon Koden EEG9100K machine (manufactured in Tomioka, Japan) using the international 10−20 system of bipolar and monopolar electrodes placement in anterior-posterior, transverse, and oblique derivation. The same neurology technician and neurologist recorded and verified the EEG, respectively, for all patients.

A possible correlation of EEG abnormality with 28-day survival was analyzed.

### 2.1. Statistical analysis

All the results and observations obtained were analyzed as per standard statistical methods using the Statistical Package for the Social Science (SPSS) version 16 IBM Corporation. Data were reported as proportions or mean±SD. Chi-square test or Fisher exact test was used for categorical variables. Normally distributed continuous variables were compared using the Student t-test (unpaired data) to analyze significant effects between the survivor and the non-survivor. *P*<0.05 was considered statistically significant.

## 3. Results

Ninety-two patients fulfilled the inclusion criteria from the database. Seventy-eight of 92 (85%) patients exhibited an abnormal EEG pattern. Fifty-three of 92 (58%) patients exhibited theta waves as the background activity, indicating advanced encephalopathy. Bilateral slowing with triphasic pattern was seen in 31 of 78 (40%) patients. Alcoholic liver disease was the most common etiology (62%) (Table 1).

**Table 1:** Demographic and EEG characteristics of study patients

Parameters	Number of patients (*n*=92) (*n* %)
Age group (years)	
21−30	5 (5)
31−40	13 (14)
41−50	26 (28)
51−60	33 (35)
61−70	13 (14)
71−80	2 (2)
Age (years)	49.94±1.1*
Sex	
Male	75 (82)
Female	17 (18)
Etiology	
Ethanol	57 (62)
Non-alcoholic Steato Hepatitis	14 (15)
Viral	12 (13)
Autoimmune	5 (6)
Cryptogenic	4 (4)
EEG findings	
Normal	14(15)
Abnormal	78 (85)
Observed EEG activity	
Background activity (*n*=92) (%)	
Alpha activity (8-10 Hz) with normal EEG pattern	24 (26)
Alpha activity (8-10 Hz) with abnormal EEG pattern	9 (10)
Theta activity (5-6 Hz) with abnormal EEG pattern	53 (58)
Delta activity (3-4 Hz) with abnormal EEG pattern	6 (6)
Abnormal EEG patterns (*n*=78) (%)	
Cerebral dysrhythmias	19 (24)
Bilateral slowing without triphasic pattern	28 (36)
Bilateral slowing with triphasic pattern	31 (40)

* Mean±standard deviation; EEG: electroencephalography

Patients with an abnormal EEG pattern had a significantly higher MELD score compared to the normal EEG group (*P*=0.02). There was no significant difference in the duration of mechanical ventilation between both the groups, but a rising trend was seen in the abnormal EEG group (*P*=0.09). Both the groups were comparable with respect to SOFA score, CTP class, serum ammonia levels, and length of ICU stay (Table 2). Patients in the normal EEG group showed a significantly better survival as compared to those with an abnormal EEG, (*P*=0.002) (Table 3).

**Table 2:** Observed variables in patients with Abnormal and Normal EEG patterns

Parameter	Abnormal EEG (78)	Normal EEG (14)	P-value*
Age*(years)	49.7±10.7	51±10.7	0.69
HE (number of patients)			
III	58	8	0.19
IV	20	6	
Tracheostomy (number of patients)	7	2	0.53
SOFA at admission*	15.6±1.7	14.9±1.5	0.13
SOFA on day of EEG*	12.6±1.7	12.5±1.6	0.84
Delta SOFA*	3±1.5	2.4±2.2	0.17
Length of ICU stay*(days)	12.3±5.6	9.9±5	0.13
Length of mechanical ventilation*(days)	10.4±5.09	8±3.4	0.09
Time to intubation*(days)	3.4±6.1	1.3±0.5	0.13
CTP*	12.6±1.4	12±1.4	0.12
MELD*	29.3±6.4	25±4.7	0.02
Serum ammonia **	232.9±118.4	202±130.6	0.38

* Mean ± standard deviation; EEG: Electroencephalography; HE: Hepatic Encephalopathy; SOFA: Sequential organ failure assessment score; CTP: Child-Turcotte-Pugh score; MELD: Model for End-Stage Liver Disease.

** Arterial ammonia in μmol/L

**Table 3:** Association of EEG findings with 28-day survival

	Outcome		*P*-value	Odds ratio (95% CI)

	Survivor	Non-survivor		
EEG				
Abnormal	23	55	0.002	5.99 (1.9-20.8)
Normal	10	4		

EEG: electroencephalography; 95% CI: 95% confidence interval

**Figure 1:**
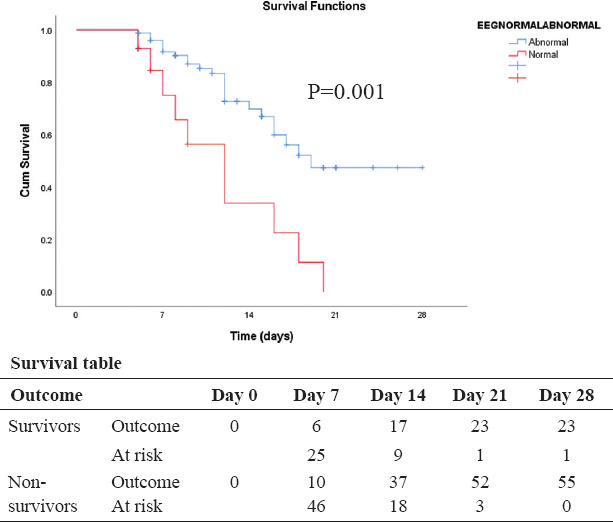
Kaplan-Meier survival curve in patients with hepatic encephalopathy grade III-IV.

The median survival was not achieved in the abnormal EEG group as shown in the Kaplan Meier’s graph in Figure 1. The mean survival duration was 15.4 days (13.6−17.2 days) in the abnormal EEG group, whereas it was 26.4 days (23.9−28.9 days) in the normal EEG group (*P*=0.001)

The sensitivity of an EEG in predicting mortality was 93.2%, with a negative predictive value of 71.4% (45.4, 88.3) and diagnostic accuracy of 63.7% (54, 72.4) (Table 4).

**Table 4:** Role of abnormal EEG in predicting mortality

Parameter	Estimate (%)	Lower - Upper 95% CIs
Sensitivity	93.2	(83.8, 97.3)
Specificity	23.3	(13.2, 37.7)
Positive predictive value	62.5	(52, 71.9)
Negative predictive value	71.4	(45.4, 88.3)
Diagnostic accuracy	63.7	(54, 72.4)

95% CI: 95% confidence interval

**Figure 2:**
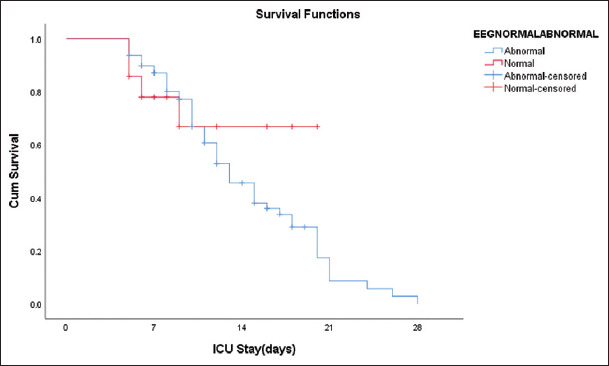
Kaplan Meier survival curve showing the ICU length of stay in patients of hepatic encephalopathy grade III-IV with normal and abnormal EEG (electro encephalography).

The median length of ICU stay of survivors with abnormal EEG was 12.9 days and of non-survivors was 11.01 days (*P*=0.47) (Figure 2).

Non survivors with an abnormal EEG had a longer ICU length of stay (p=0.012), a higher SOFA score at admission (p=0.008), higher SOFA score on the day of EEG recording (p=0.006), a higher MELD score (p=0.021), and required more days on mechanical ventilation (p=0.01) (Table 5).

**Table 5:** Comparison of survivors versus non-survivors with secondary outcome measures

	Non survivor (59)	Survivor (33)	*P*-value^#^
Age (years)	49.42±11.02	50.85±9.98	0.54
Sex male	51	24	0.104
Abnormal EEG pattern			
Cerebral dysrhythmias	5	14	0.17
Bilateral slowing without triphasic pattern	7	21	0.20
Bilateral slowing with triphasic pattern	13	18	0.38
SOFA at admission^#^	15.85±1.71	14.88±1.57	0.009
SOFA on day of EEG^#^	12.95±1.64	11.94±1.67	0.006
D SOFA	2.90±1.51	2.94±1.87	0.91
Time to intubation*	3.63±6.51	2.97±3.81	0.7
Length of ICU stay^#^	12.24±6.08	11.30±4.41	0.47
Length of mechanical ventilation*	10.93±5.43	8.33±3.34	0.012
CTP^#^	12.71±1.39	12.21±1.39	0.1
MELD^#^	29.75±6.48	26.64±5.7	0.024
S NH3*	243.66±127.65	200.67±101.18	0.11

# Pearson chi-squared p-value was utilized.

* p value was calculated using Mann–Whitney test. Mean ± standard deviation is reported for Chi-square and Mann–Whitney tests. EEG: electroencephalography; HE: hepatic encephalopathy; SOFA: sequential organ failure assessment score; CTP: Child-Turcotte-Pugh score; MELD: model for end-stage liver disease; S NH3: serum ammonia

## 4. Discussion

In this retrospective study, majority of patients with HE had EEG abnormalities (85%). The presence of an abnormal EEG was associated with low survival.

A high MELD score on admission was associated with abnormal EEG findings and mortality. HE is not included in the MELD score, but combining the severity of HE with the MELD score provides the best prediction of mortality [15]. However, among them who are more likely to die is not well studied.

In our study, 58% patients exhibited background slowing with theta activity while in few cases it transitioned further to delta activity (6%). The peculiar and earliest characteristic of HE is the loss of alpha rhythm frequency, which gradually leads to the onset of slower rhythms [12]. Marchetti *et al*. showed that patients with overt HE had much slower mean frequencies than patients with minimal HE [16]. Slowing of frequency was accompanied with a triphasic pattern in 40% of our patients. Triphasic waves are medium to high amplitude (100-300 mv) waves with a frequency of 1.5-2.5 Hz, which have three phases without an extra spike/polyspike component. These triphasic waves were initially thought to be pathognomonic of HE [17,18], but later, they were observed in other metabolic encephalopathies as well [19-21]. Contrarily, there has been reports of hepatic encephalopathy presenting as generalized convulsive status [22,23]. Therefore, it is important to consider the possibility of non-convulsive status epilepticus, especially in high grade HE, non-responsive to anti coma measures [24-26]. Cerebral dysrhythmias in EEG are often reported as either focal or generalized spikes/sharp wave discharges, similar to epileptiform discharges. We found an incidence of 24%, for cerebral dysrhythmias in our study.

Despite notable differences in acquisition and analytical methods, consistent EEG findings in HE include progressive slowing of frequency from alpha rhythm to theta and delta rhythm. Studies have proven that abnormal EEG patterns, either triphasic waveform or epileptiform discharges indicate poor prognosis in terms of survival [7,14,27-30]. The present study included patients with advanced HE (Grade III-IV), which is a poor prognostic indicator for survival [31-33]. However, patients with advanced HE and a normal EEG had a significant better 28-day survival compared to those with abnormal EEG patterns. Therefore, EEG proved to be a reliable prognostic indicator in advanced HE.

In our study, non survivors with an abnormal EEG had longer ICU length of stay, a higher SOFA score at admission, higher SOFA score on the day of EEG recording and a higher MELD score. Hence, EEG abnormalities in patients with high MELD score and higher grades of HE have a higher prognostic benefit. As our sample size was small, the correlation of the EEG pattern with mortality cannot be commented on.

The specificity of an EEG in predicting mortality was 23.3% (13.2, 37.7) with a negative predictive value of 71.4% (45.4, 88.3) and diagnostic accuracy of 63.7% (54, 72.4). Sara Montagnese *et al*. concluded that MELD-EEG had higher prognostic accuracy in predicting 12- and 18-month mortality compared to MELD (p 0.016 and p 0.018, respectively) and the addition of an automatically obtained EEG-based index improved the prognostic accuracy of the MELD score [34].

Dasgupta *et al*. concluded that EEG is a low-cost diagnostic tool which can be easily performed in minimal HE and has a positive correlation with advanced CTP-Class, higher MELD scores and high ammonia levels [35]. However, the correlation of EEG in HE Grade III-IV and its prognostic implication is lacking in the literature.

Although the grading of severity of EEG alterations in HE can be evaluated based on visual pattern recognition, this method has shown to have limited reliability [36] semi-quantitative analysis for base frequency [27] or quantitative spectral analysis [37,38] with or without brain mapping needs to be included in protocols [39,40].

In our study, we excluded all the patients who had encephalopathy secondary to metabolic derangements other than liver disease. None of the patients with epileptiform discharges had clinical evidence of seizure activity. However, the possibility of non-convulsive status epilepticus cannot be ruled out in these cases, as its diagnosis ideally requires continuous EEG monitoring [41,42], which was not employed in our study.

Drawbacks of our study are its retrospective nature and absence of continuous EEG monitoring.

## 5. Conclusion

The results of this study demonstrate that EEG analysis plays a significant role in evaluation of advanced HE in critically ill patients with liver cirrhosis. The most common EEG finding in HE Grade III-IV is bilateral slowing, with triphasic pattern being common. Cerebral dysrhythmias are also seen in few cases, which may be an indicator of non-convulsive status epilepticus (NCSE). This subset of patients might be candidates for continuous EEG monitoring. This study reinforces the prognostic significance of EEG abnormalities in critically ill cirrhotic patients with advanced HE.

## References

[1] Rothstein JD, Herlong HF (1989). Neurologic Manifestations of Hepatic Disease. Neurol Clin.

[2] D'Amico G, Garcia-Tsao G, Pagliaro L (2006). Natural History and Prognostic Indicators of Survival in Cirrhosis:A Systematic Review of 118 Studies. J Hepatol.

[3] Romero-Gomez M, Montagnese S, Jalan R (2015). Hepatic Encephalopathy in Patients with Acute Decompensation of Cirrhosis and Acute-on-chronic Liver Failure. J Hepatol.

[4] Stewart CA, Malinchoc M, Kim WR, Kamath PS (2007). Hepatic Encephalopathy as a Predictor of Survival in Patients with End-Stage Liver Disease. Liver Transpl.

[5] Cordoba J, Ventura-Cots M, Simon-Talero M (2014). Characteristics, Risk Factors, and Mortality of Cirrhotic Patients Hospitalized for Hepatic Encephalopathy with and Without Acute-on-Chronic Liver Failure (ACLF). J Hepatol.

[6] Kaplan PW, Rossetti AO (2011). EEG Patterns and Imaging Correlations in Encephalopathy:Encephalopathy Part II. J Clin Neurophysiol.

[7] Foley JM, Watson CW, Adams RD (1950). Signiﬁcance of the Electroencephalographic Changes in Hepatic Coma. Trans Am Neurol Assoc.

[8] Vilstrup H, Amodio P, Bajaj J, Cordoba J, Ferenci P, Mullen KD (2014). Hepatic Encephalopathy in Chronic Liver Disease:2014 Practice Guideline by the American Association for the Study of Liver Diseases and the European Association for the Study of the Liver. Hepatology.

[9] Saxena N, Bhatia M, Joshi YK, Garg PK, Dwivedi SN, Tandon RK (2002). Electrophysiological and Neuropsychological Tests for the Diagnosis of Subclinical Hepatic Encephalopathy and Prediction of Overt Encephalopathy. Liver.

[10] Hartmann IJ, Groeneweg M, Quero JC, Beijeman SJ, de Man RA, Hop WC (2000). The Prognostic Significance of Subclinical Hepatic Encephalopathy. Am J Gastroenterol.

[11] Koziarska D, Wunsch E, Milkiewicz M, Wojcicki M, Nowacki P (2013). Milkiewicz Mini-Mental State Examination in Patients with Hepatic Encephalopathy and Liver Cirrhosis:A Prospective, Quantiﬁed Electroencephalography Study. BMC Gastroenterol.

[12] Parsons-Smith BG, Summerskill WH, Dawson AM, Sherlock S (1957). The Electroencephalograph in Liver Disease. Lancet.

[13] Montagnese S, Amodio P, Morgan MY (2004). Methods for Diagnosing Hepatic Encephalopathy in Patients with Cirrhosis:A Multidimensional Approach. Metab Brain Dis.

[14] van der Rijt CC, Schalm SW (1985). Quantitative EEG Analysis and Survival in Liver Disease. Electroencephalogr Clin Neurophysiol.

[15] Bajaj JS, O'Leary JG, Tandon P, Wong F, Garcia-Tsao G, Kamath PS (2017). Hepatic Encephalopathy is Associated with Mortality in Patients with Cirrhosis Independent of Other Extrahepatic Organ Failures. Clin Gastroenterol Hepatol.

[16] Marchetti P, D'Avanzo C, Orsato R, Montagnese S, Schiff S, Kaplan PW (2011). Electroencephalography in Patients with Cirrhosis. Gastroenterology.

[17] Bickford RG, Butt HR (1955). Hepatic coma:The Electroencephalographic Pattern. J Clin Invest.

[18] Brenner RP (2005). The Interpretation of the EEG in Stupor and Coma. Neurologist.

[19] Kaplan PW (2004). The EEG in Metabolic Encephalopathy and Coma. J Clin Neurophysiol.

[20] Sundaram MB, Blume WT (1987). Triphasic Waves:Clinical Correlates and Morphology. Can J Neurol Sci.

[21] Young GB, Bolton CF, Austin TW, Archibald YM, Gonder J, Wells GA (1990). The Encephalopathy Associated with Septic Illness. Clin Invest Med.

[22] Ficker DM, Westmoreland BF, Sharbrough FW (1997). Epileptiform Abnormalities in Hepatic Encephalopathy. J Clin Neurophysiol.

[23] Tanaka H, Ueda H, Kida Y, Hamagami H, Tsuji T, Ichinose M (2006). Hepatic Encephalopathy with Status Epileptics:A Case Report. World J Gastroenterol.

[24] Eleftheriadis N, Fourla E, Eleftheriadis D (2003). Status Epilepticus as a Manifestation of Hepatic Encephalopathy. Acta Neurol Scand.

[25] Newey CR, George P, Sarwal A, So N, Hantus S (2018). Electro-Radiological Observations of Grade III/IV Hepatic Encephalopathy Patients with Seizures. Neurocrit Care.

[26] Jo YM, Lee SW, Han SY, Baek YH, Ahn JH, Choi WJ (2015). Nonconvulsive Status Epilepticus Disguising as Hepatic Encephalopathy. World J Gastroenterol.

[27] Amodio P, Pellegrini A, Ubiali E, Mathy I, Piccolo FD, Orsato R (2006). The EEG Assessment of Low-Grade Hepatic Encephalopathy:Comparison of an Artiﬁcial Neural Network-Expert System (ANNES) Based Evaluation with Visual EEG Readings and EEG Spectral Analysis. Clin Neurophysiol.

[28] Montagnese S, DeRui M, Schiff S, Ceranto E, Valenti P, Angeli P (2015). Prognostic Beneﬁt of the Addition of a Quantitative Index of Hepatic Encephalopathy to the MELD Score:The MELD-EEG. Liver Int.

[29] Wszolek ZK, Aksamit AJ, Ellingson RJ, Sharbrough FW, Westmoreland BF, Pfeiffer RF (1991). Epileptiform Encephalographic Abnormalities in Liver Transplant Recipients. Ann Neurol.

[30] Sutter R, Stevens RD, Kaplan PW (2013). Significance of Triphasic Waves in Patients with Acute Encephalopathy:A Nine-Year Cohort Study. Clin Neurophysiol.

[31] Tapper EB, Aberasturi D, Zhao Z, Hsu CY, Parikh ND (2020). Outcomes after Hepatic Encephalopathy in Population-Based Cohorts of Patients with Cirrhosis. Aliment Pharmacol Ther.

[32] Ezaz G, Murphy SL, Mellinger J, Tapper EB (2018). Increased Morbidity and Mortality Associated with Falls among Patients with Cirrhosis. Am J Med.

[33] Bustamante J, Rimola A, Ventura PJ, Navasa M, Cirera I, Reggiardo V (1999). Prognostic Significance of Hepatic Encephalopathy in Patients with Cirrhosis. J Hepatol.

[34] Montagnese S, de Rui M, Schiff S, Ceranto E, Valenti P, Angeli P (2015). Prognostic Benefit of the Addition of a Quantitative Index of Hepatic Encephalopathy to the MELD Score:The MELD-EEG. Liver Int.

[35] Dasgupta A, Debbarma A, Choudhury SK (2019). Evaluation of the Role of Electroencephalography in the Early Diagnosis of Minimal Hepatic Encephalopathy in Patients with Cirrhosis of Liver. J Evid Based Med Healthc.

[36] Amodio P, Marchetti P, Del Piccolo F, de Tourtchaninoff M, Varghese P, Zuliani C (1999). Spectral Versus Visual EEG Analysis in Mild Hepatic Encephalopathy. Clin Neurophysiol.

[37] Amodio P, Del Piccolo F, Petteno E, Mapelli D, Angeli P, Iemmolo R (2001). Prevalence and Prognostic Value of Quantiﬁed Electroencephalogram (EEG) Alterations in Cirrhotic Patients. J Hepatol.

[38] Guerit JM, Amantini A, Fischer C, Kaplan PW, Mecarelli O, Schnitzler A (2009). Neurophysiological Investigations of Hepatic Encephalopathy:ISHEN Practice Guidelines. Liver Int.

[39] Sagalés T, Gimeno V, de la Calzada MD, Casellas F, Macià MD, Soriano MV (1990). Brain Mapping Analysis in Patients with Hepatic Encephalopathy. Brain Topogr.

[40] Kullmann F, Hollerbach S, Lock G, Holstege A, Dierks T, Schölmerich J (2001). Brain electrical Activity Mapping of EEG for the Diagnosis of (Sub)clinical Hepatic Encephalopathy in Chronic Liver Disease. Eur J Gastroenterol Hepatol.

[41] Kubota Y, Nakamoto H, Egawa S, Kawamata T (2018). Continuous EEG Monitoring in ICU. J Intensive Care.

[42] Abend NS, Dlugos DJ, Hahn CD, Hirsch LJ, Herman ST (2010). Use of EEG monitoring and Management of Non-Convulsive Seizures in Critically Ill Patients:A Survey of Neurologists. Neurocrit Care.

